# In search of member needs in coworking spaces

**DOI:** 10.1007/s11846-022-00546-4

**Published:** 2022-04-07

**Authors:** Erik Rådman, Erik Johansson, Petra Bosch-Sijtsema, Hendry Raharjo

**Affiliations:** Division of Service Management and Logistics, Department of Technology Management and Economics, Chalmers University of Technology, 41296 Gothenburg, Sweden

**Keywords:** Coworking space, Member need, Self-determination theory, Tensions

## Abstract

Coworking spaces represent a new trend for future workplaces. As more building owners are interested in running coworking space businesses, it becomes important to understand both why a potential member chooses one space over another and how to keep existing members at a coworking site. A sound understanding of member needs can make a difference. Unfortunately, very few studies have been conducted as to understanding a member’s basic needs in coworking spaces. In this paper, we aim to identify member’s needs in three coworking spaces in Sweden. Participant observations, immersion, and interviews were used for data collection. The member needs are categorized and structured through the lens of self-determination theory. In total, we uncovered, formulated, and categorized 21 member needs. We found that the fulfillment of one need may lead to the inhibition of another, thus creating tensions between and within coworking members. This research contributes to the literature by addressing the importance and definition of member needs for coworking as well as the created tensions related to these needs, which have been lacking in coworking studies.

**Supplementary information:**

The online version contains supplementary material available at 10.1007/s11846-022-00546-4.

## Introduction

The number of coworking spaces increased worldwide from 8,900 to 2015 to 18,700 in 2018 (Deskmag 2019), and it is forecast to reach about 42,000 in 2024 (Coworking Resources & Coworker 2020). Coworking spaces provide an answer to the increasing demand for a more flexible workplace and growing work-individualization, such as self-employment, freelance, or on-demand work. It is considered as a sharing economy practice that can have a positive impact on entrepreneurial outcomes (Bouncken & Reuschl 2018) and may contribute to a more sustainable society (Mi & Coffman 2019).

As more building owners become interested in running a coworking space or transforming their existing spaces into a coworking space, it becomes important to understand why a potential member selects one coworking space over another, as well as how to keep existing members at a coworking site. A sound understanding of member needs can make a difference. However, there have been few studies on understanding member needs in coworking spaces. The closest related research is on analyzing user preferences for coworking spaces (Weijs-Perrée et al. 2019; Appel-Meulenbroek et al. 2020). They showed that accessibility and contract options are among the most important issues to resolve when choosing a specific coworking space. Although these findings are important, they give less insight into what coworking providers can do to improve the quality of their spaces and maintain their coworking members. For example, if the location is not in the city center or accessible by public transport, efforts to improve its accessibility may not be cost efficient or even feasible. Furthermore, knowing that the contract options are important may not be useful since this is the very reason why people choose coworking spaces over conventional offices. If several coworking spaces are located strategically and with equally flexible contracts, they must find other ways to differentiate themselves. Understanding member preferences is useful when trying to attract potential members. However, to keep the existing members, we argue that one should go a step further, that is, by understanding the member’s basic needs.

Using the perspective of quality management (Bergman & Klefsjö 2010), the job-to-be-done approach (Ulwick & Bettencourt 2008), and self-determination theory (Deci & Ryan 2002), we define a member need as a description of a job, which addresses innate psychological nutriments that are essential for ongoing psychological growth, integrity, and well-being, to be fulfilled by the product or service. Although the coworking literature has discussed motivation and user preferences, little research exists into the basic psychological needs of the members. Basic needs can be related to psychological motivation theory in which three universal needs are identified: autonomy, relatedness, and competence. Psychological theory concerning needs has found that the cultural and social environment play an important part in supporting or hindering performance and well-being (Deci & Ryan 2000, 2014; Ryan & Deci 2002), as well as the physical environment (Sjöblom et al. 2016). An insight into how the workplace environment supports or hinders the fulfilment of needs becomes relevant for building owners to maintain their members’ satisfaction with the coworking place and offered services.

In this paper, we aim to identify member needs in three coworking spaces in Sweden. The Swedish coworking market is dominated by large-scale actors. As of 2020, the five largest coworking space providers constitute 50% of the coworking market in the capital of Sweden (Fastighetsägarna 2020). Our starting point are the providers who are keen to know their coworking members and their needs to support performance and well-being in the coworking space. Based on participant observations, immersion, and in-depth interviews, we formulated these needs with the help of self-determination theory as a theoretical lens (Deci & Ryan 1985). The uncovered needs would provide a starting point for coworking space providers to improve their services and to create competitive advantages through various innovative solutions or service offerings.

The article is structured as follows. Section 2 provides a short overview of state-of-the-art coworking research focusing on what coworking spaces are and why people co-work, followed by a theoretical background on self-determination theory and the distinction between needs, motivations, and preferences. Section 3 describes the research method and how we collected and analyzed the data. The empirical results are provided in Sect. 4. The discussions of the results with respect to the existing research along with their managerial implications are described in Sect. 5. Lastly, Sect. 6 concludes the research.

## Theoretical background

### What are coworking spaces?

The aspect that unites scholarly descriptions of coworking is the aspect of co-location of unaffiliated knowledge workers (Bilandzic & Foth 2013; Capdevila 2013; Gandini 2015; Parrino 2015). Spinuzzi (2012) described coworking spaces as *“open-plan office environments in which they [mobile professionals] work alongside other unaffiliated professionals.”* There are numerous other aspects discussed, for example, a *“community-like environment”* that helps the coworking users in their networking efforts within and outside of the coworking space (Rese et al. 2021), social learning, peer collaboration, creativity (Bilandzic & Foth 2013), knowledge sharing (Capdevila 2013; Parrino 2015), and an atmosphere or lifestyle (Moriset 2014). The growth of coworking spaces in urban and rural settings is also said to facilitate entrepreneurship (Bouncken et al. 2020a). Several authors have also identified different types of coworking spaces. Initially, coworking was primarily focused on start-ups, freelancers and entrepreneurs, but in recent years, larger and established firms are also using coworking spaces (Kraus et al. 2022, Orel & Bennis 2021). Bouncken et al. (2018) classified four different types of coworking (i.e., corporate, open corporate, consultancy, and independent coworking spaces) and identified tensions regarding value creation and appropriation related to coopetition. Orel and Bennis (2021) developed a taxonomy of four different coworking models: (a) the individual-purposed space in which freelancers and location independent professionals work alongside, (b) the creation-purposed space focusing on jointly creating like a makerspace, (c) a group-purposed space focusing on teams often of larger firms, and (d) a startup-purposed coworking space.

### Why co-work?

Coworking members are often portrayed as freelancers, entrepreneurs, knowledge workers, nomadic workers, self-employed workers (Spinuzzi 2012; Waters-Lynch & Potts 2017; Vidaillet & Bousalham 2018; Merkel 2019; Kraus et al. 2022). But it is also common for coworking members to be employees of organizations (van Dijk 2019; Bouncken et al. 2021).

Several attempts have been made to understand *why* people join coworking spaces through identification of motivations and user preferences. Based on previous coworking research over the years, Table 1 shows the various reasons people join coworking spaces, which include social interaction, being part of a community, and the wish to escape the social isolation of work.

**Table 1:** Why people co-work

	Spinuzzi (2012)	Gandini (2015)	Ross & Ressia (2015)	Brown (2017)	Garret et al. (2017)	Jakonen et al. (2017)	Butcher (2018)	Clifton et al. (2019)	van Dijk (2019)	Weijs-Perrée et al. (2019)	Appel-Meulenbroek et al. (2020)
Social Interaction	x		x				x	x		x	x
Being part of a community	x				x	x	x			x	x
The wish to escape the social isolation of work	x	x			x	x		x			
Accessing a professional work environment	x			x							
Acquire a professional status associated with the space		x							x		
Networking opportunities		x	x	x				x	x	x	x
Affordable accommodation			x					x		x	
Flexibility	x		x							x	

Several benefits of coworking have been proposed, such as providing a sense of community (Parrino 2015), knowledge exchange (Capdevila 2013), innovation (Yang et al. 2019), work satisfaction, creativity, entrepreneurship (Bouncken & Aslam 2019), and productivity (Bueno et al. 2018). A more recent study showed that coworking may help improve equality within entrepreneurship and benefit those who need help the most (Howell, 2022). Although often portrayed in a good light, not all promises of coworking spaces are fulfilled (Nakano et al. 2020). Cocreation of value is not always guaranteed (Goermar et al. 2021) and formal collaborations between members do not necessarily take place (Wijngaarden et al. 2020). Tensions can arise from fair acknowledgement of work when sharing resources in a collaborative value-creation process (Waters-Lynch & Duff 2019). Knowledge leakage because of opportunistic members can impair learning and performance, causing reduction of trust and community building (Bouncken & Reuschl 2018). Bilandzic and Foth (2013) find barriers for social learning since users find it hard to identify or approach each other – remaining unaware of each other’s expertise. Parrino (2015) notes that some users avoid, or neglect encounters due to a primary focus on their work. Furthermore, co-location can lead to noise and unavoidable interactions which might lower the work satisfaction of the coworking members (Bouncken et al. 2020b). Aslam et al. (2021) highlights the importance of materiality’s role on the social phenomenon in coworking which can lead to both instrumental and detrimental outcomes for entrepreneurs.

### Self-determination theory (SDT)

SDT has been used extensively in organizational psychology to promote wellness and productivity in organizations (Deci et al. 2017). SDT is a motivational theory that views needs as human universals and essential psychological nutrients (Deci & Ryan 2014). A need is defined as an “*innate psychological nutriments that are essential for ongoing psychological growth, integrity, and well-being*” (Deci & Ryan 2002: p. 229).

For psychological health and performance, the degree of satisfying the needs becomes important. When basic psychological needs are satisfied, individuals are more autonomously motivated, and they behave with a sense of volition, willingness and choice as opposed to being unmotivated or controlled. According to Deci and Ryan (2014), autonomous motivation consists of both intrinsic motivation (performing something with enjoyment and interest) and fully internalized extrinsic motivation (performing something volitionally because of its personal importance or value).

We use the SDT lens to unfold and categorize the identified user needs. SDT encompasses three needs: relatedness, autonomy, and competence (Deci & Ryan 1985, 2000). *Relatedness* is about the desire to be connected to others, caring for and being cared for by others, having a sense of belongingness with other individuals and a community (Ryan & Deci 2002). The need for *autonomy* is acting from interest and integrated values and experiencing one’s behavior as an expression of self. In a workplace context, people with a high degree of autonomy tend to find ways of satisfying the needs of the other two dimensions as well. Autonomy should not be confused with independence. *Competence* is about feeling reflective in the interactions with the environment, expressing capacities, and experiencing opportunities (Ryan & Deci 2002). Fulfilling the needs for relatedness, autonomy, and competence leads to intrinsic motivation, general well-being, and psychological growth. However, not being able to fulfill the needs for relatedness, autonomy, and competence can lead to frustration, subpar performance, and sometimes psychological distress (Deci et al. 2017).

The need for relatedness and autonomy are often complementary. However, at times, sub-optimal environmental conditions can create tension and conflict between the two, for example, when the internal values of a person differ considerably from those of the group (Deci & Ryan 2000). Deci and Ryan (2014) mention how the implications of the work environment affect people’s performance and well-being and how a work environment supporting people’s needs has positive results for performance. While SDT has been used in different areas, research on using SDT in relation to physical environments has been scarce (Sjöblom et al. 2016). The work environment can be viewed as social, cultural, and a physical environment that can support the fulfillment of the individual’s psychological needs.

### Member needs, motivations, and preferences

**Fig. 1:**
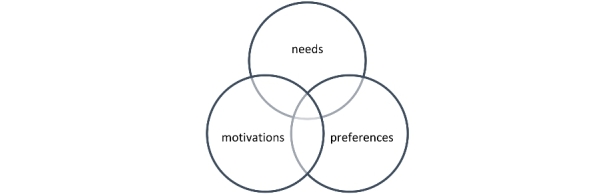
Needs, motivations, and preferences

The concept of needs used in SDT is different from the broader concepts of personal motives, desires, or endeavors (Deci & Ryan 2014). Not all motives and desires fulfill the basic psychological needs; they can even be distracting. One example of why people join a coworking space is ‘to acquire a professional status associated with the space’ (van Dijk 2019; Gandini 2015). Is this a need, a motivation, or a preference? We argue that this is a motivation, not a need or a preference. The need could be to gain trust from the clients. Another example is ‘the desire to be part of a community’ (Spinuzzi 2012; Garret et al. 2017; Jakonen et al. 2017; Weijs-Perrée et al. 2019), which can be both a need and a motivation, but not a preference. Examples of preferences are ‘accessibility’ and ‘contract options’ (Weijs-Perrée et al. 2019; Appel-Meulenbroek et al. 2020). However, these are not needs. In brief, these three entities have some overlap, but they are not the same (see Fig. 1).

We differentiate between these three terms in spite of the overlap. As mentioned previously, we define a member need as ‘*a description of a job, which addresses innate psychological nutriments that are essential for ongoing psychological growth, integrity, and well-being, to be fulfilled by the product or service.*’ For the definition of motivation, we follow Weinstein and Ryan (2010) who defined it as “*the quality of experience that energizes behavior*” (p. 223) For preference, we refer to it as “*a greater liking for one alternative over another or others*” as in the *Oxford Dictionary of English*. In this case, preferences are more solution-focused than need-focused.

A common practice in many fields when focusing on understanding users’ satisfaction is to carry out surveys. Such surveys are either solution-focused or retrospective. Griffin and Hauser (1993) have pointed out that corporations run the risk of developing counterproductive product or service features when basing their decisions on collected user satisfaction data. In the coworking context, suppose the provider runs a member satisfaction survey on breakfast meetings during the last year. The members can effortlessly come up with comments on the dishes served in a common breakfast meeting, instead of seeing how such an activity supports the need for the members to belong to the community. Improving the common breakfast meeting dishes may not be effective since another solution to the same need could be more effective, such as a potluck lunch or common sports activities. Ulwick and Bettencourt (2008) have argued that when asking users what they prefer, they often state that they need more of what the product or service already provides, mostly focusing on the solutions or offerings instead of the job a user wants to accomplish when using that product or service.

To sum up, understanding a member’s motivations and preferences is critical for attracting potential members, that is, why they choose one place over another. However, to retain the existing members so that they enjoy and thrive, one should go a step further by understanding the members’ needs. Hence, the coworking providers can take more deliberate actions to satisfy those needs and stand out from the competition by improving existing features and/or solutions or creating new ones through innovation (Griffin & Hauser 1993; Cristiano et al. 2000).

## Research methods

To understand the member needs, we applied an abductive approach (as in Dubois & Gadde 2002), in which inductive and deductive approaches follow one another. The study started more inductively with observations and later on interview questions were supported by the theoretical framework of STD. Ulwick and Bettencourt (2008) and Griffin and Hauser (1993) have stated that best-practice methods for identifying user/member needs are qualitative methods such as interviews and “visits” (participant observations). We employed semi-structured interviews, participant, and non-participant observations to generate data, which we used together with secondary data from the coworking spaces. A research team of four researchers (including the two researchers conducting interviews and observations) had weekly meetings to discuss the observations and findings. The meetings also served the purpose of guiding the continued process of data collection and iteratively provided peer researchers’ perspectives on the data during analysis.

### Data collection

**Research context**.

The researchers had access, as members, to three coworking spaces in Gothenburg, Sweden, for six months (January–June 2020). The coworking spaces in the study were owned by different real estate companies and were similarly priced in the higher range of the Swedish coworking market (see Table 2). Overall, there were three types of membership. First, a *lounge membership* allowed access to the common area of the coworking space. Second, a *flex membership*, in addition to the common area, gave access to an area with ergonomic chairs and desks. Third, a *private office membership*, which gave access to a private office and the common area. Amenities and service functions were included in all three memberships. One of the coworking spaces had more members from larger organizations (see Table 2) than the other two spaces, it could be whole departments from a large organization or a couple of employees living in the same city. Large organizations have 250 or more employees, medium organizations have between 50 and 249 employees, and small organizations have between 10 and 49 employees. The cases show a combination of individual-purposed coworking spaces with freelancers and location independent workers as well as group-purposed coworking spaces in which larger companies and teams are located but still form a community across non-affiliated entities (Orel & Bennis 2021).

**Table 2:** Research context

	Coworking space 1	Coworking space 2	Coworking space 3
Location	City centre	Just outside the city centre	City centre
Number of members	200–300	50–100	< 50
Price	Mid-high	Mid-high	Mid-high
Main type of member organization size	Medium/Large	Small	Small/Medium
Membership types	Lounge, flex, private office	Lounge, private office	Lounge, private office

**Observations**.

During a period of six months, the researchers spent time as members at the three coworking spaces, performing participant and non-participant observations. Two researchers were present most days of the week, usually during normal office hours. Occasionally, the researchers performed observations during early mornings, late evenings, and weekends to gain an understanding of the space during different times and hours in the week. Similarly, an effort was made to experience as many rooms and seating areas of the settings as possible.

Over 900 h of observations were performed at the coworking spaces. Guba and Lincoln (1994) argue that the trustworthiness of a study increases if considerable time is spent in the setting. The researchers participated in various events, such as member breakfasts, after works, face-to-face and remote seminars, and remote workouts. A workshop was initially planned for one of the lunch events but had to be canceled due to the ongoing Covid-19 pandemic. Note that there were no lockdowns in Sweden during the pandemic, but events with more than 50 people were not allowed.

The observations gave rich insights into the coworking space and its members. To give one example, it was observed that many members gave visitors a tour of the coworking space, which was not mentioned in the interviews until the researchers explicitly asked about it.

An overt observational approach was applied; the researchers introduced themselves and the research project when given the opportunity. Field notes were taken in a project diary where novel experiences, thoughts, and observations were noted and the setting, date, and time were recorded in accordance with Clancey (2006). Also, notes and photos were taken of seating arrangements, walking paths, and the behavior of members. Furthermore, we observed interactions between members in different settings as well as members working individually on a computer or communicating in phone/video calls. Efforts were also made to keep observational notes descriptive and separate from the researcher’s thoughts or experiences.

**Interviews**.

In addition to the observations, we also held several interviews. We held three interviews with the hosts of the spaces to gain insight into each space and its members. In addition to these interviews, we held thirteen in-depth semi-structured interviews with the main purpose of identifying member needs as well as many shorter discussions with many members during the observations in all three coworking spaces (the shorter discussions were reported in the observational notes). For the in-depth interviews, members from all three coworking spaces were interviewed, with each interview taking from 45 min to an hour and a half. A purposive sampling approach was applied to cover a diverse group of interviewees with respect to characteristics such as age, gender, occupation, tenure, employment time, and the size of the employer’s organization (Table 3).

**Table 3:** Member interviews

*Gender*	*Age*	*Profession*	*Days per week at the space*	*Employment organization*
Male	25–34	Sales Manager	5	Large organization
Male	25–34	Entrepreneur, Business Developer	6	Self Employed
Male	25–34	Corporate Development Manager	5	Large organization
Female	35–44	Business Developer, HR	2–3	Small Organization
Male	25–34	Data Engineer	3	Small Organization
Female	25–34	Solution Engineer	1–2	Large Organization
Female	35–44	Entrepreneur, Process Leader	4–5	Self Employed
Male	45–54	Manager, Business Developer	4	Large Organization
Male	45–54	Entrepreneur, Business Developer	5	Self Employed
Male	55–64	Entrepreneur, Business Developer	4	Self Employed
Female	25–34	Management Consultant	1–2	Medium Organization
Female	55–64	Entrepreneur, HR	3	Self Employed
Female	45–54	Regional Manager	5	Large Organization

In Table 3, interviewees were also selected to represent different sizes of organizations in the coworking space, in which large organizations have 250 or more employees, medium organizations have between 50 and 249 employees, and small organizations have between 10 and 49 employees. The interview process stopped when we thought our sample was representative enough and additional interviews no longer provided new insights. With our interviews, conversations, observations, participant observations, and secondary data, we felt we had enough material to understand the member needs. The interviews consisted of two parts: the first part had two activities (inspired by design thinking) and the second part was a semi-structured interview.

For the first part and first activity, interviewees were asked to narrate a day in their lives while mapping their energy levels corresponding to that particular day. This activity provided a basic understanding of members’ workdays, how and when they travelled to the space, and to what extent the members stayed within the space during their workday. The second activity contained a photo-elicitation. Members were asked to select three pictures that they associated with something that made their work easier by being a member at a coworking space, followed by one picture that made it frustrating. With each choice, the member was asked to clarify and explain why that picture was chosen, after which follow-up questions were asked. The narration of a workday and the photo-elicitation provided deep insights into how members utilized the space, what parts they used, and their thoughts about other members.

After the photo-elicitation, the second part of the interview was performed in a semi-structured matter based on an interview guideline. By focusing on the job that members try to accomplish, the researchers attempted to illuminate met and unmet needs (Ulwick & Bettencourt 2008). The interview guide was iteratively updated to explore emergent themes and needs. The application of self-determination theory was incorporated in some of the interview questions to gain more insight into the psychological needs of the members. Questions used in earlier research on SDT (e.g., Van den Broeck et al. 2010; Deci and Ryan 2000) were the inspiration for some of the interview questions and the questions were tailored to a coworking setting. Interview questions focused on topics like challenges, relation to others, feeling of belonging, can you be yourself, when do you feel productive, and motivation. All interviews were recorded and transcribed with permission from the interviewees. Most interviews were held in person, and some were held via a video conferencing tool.

**Secondary data**.

The researchers were given access to customer satisfaction surveys, email communication, booking apps and space usage statistics.

### Data analysis

Observations and interviews were coded with NVivo and grouped in similar parent nodes and themes. The main method for grouping the data was through affinity mapping inspired by the Kawakita-Jiro method (Scupin 1997). The affinity mapping approach is a visual approach for analyzing, categorizing, and coding data, which was used for the interviews as well as the observational material, photographs, and the shorter conversations held with members. In the first step, observations and statements by the interviewees were written on separate Post-it notes. Post-it notes with similar themes were grouped together and categories started to emerge. Different types of categorizations were tried to reach new insights in an iterative manner as more themes and categories emerged. This process was started during the data collection phase. The weekly meetings with the research team provided opportunities for discussion of emergent themes and consulting the literature.

The next part of the analysis process involved looking at the data through the lens of SDT. The member needs were formulated following affordance theory. Affordance theory is predominantly used in product, spatial, and interaction design (Norman 1988; Gaver 1996). Affordance theory was used mostly in the form of “*to be able to*…”. When the uncovered themes had been grouped into final categories of needs, these needs were in turn grouped into the three basic psychological needs of SDT: autonomy, relatedness, and competence. A closer look at the identified member needs reveals that some needs are more closely related to each other than the others. We therefore created five overarching categories to create a structure for the SDT-categorized needs.

## Results

We uncovered a total of 21 member needs viewed through an SDT lens. Five different categories of needs emerged from the data (see Table 4) and the needs are discussed in more detail below.

**Table 4:** The needs of the coworking members

*Category*	*SDT*	*Need*
Social needs	Relatedness	To belong to a community
		To have a workplace that gives you energy
		To be noticed and feel welcome at one’s workplace
	Autonomy	To be in control of social interactions
		To be able to be transparent
Business networking	Relatedness	To have your workplace leave a good impression on guests
	Autonomy	To be able to market one’s company
		To know who the other members are
	Competence	To cooperate with relevant actors
		To meet people that can lead to business opportunities
Knowledge exchange	Competence	To learn new things from peers and events
		To be able to receive help or input from others
		To be able to share knowledge
Productivity	Autonomy	To be able to focus on work activities
		To have interactions without disturbing others
		To be able to choose a suitable work area
		To be able to manage confidential information securely
	Competence	To feel an increased productivity from one’s workplace
		To be able to focus on the core business
		To be able to work smoothly without technical disruptions
Physical well-being	Autonomy	To be healthy

### Social needs

Social needs encompass needs related to social interaction between individuals or being present at a place where social interactions take place. It is also about being able to balance social interaction with work, without being seen as uninterested by others when prioritizing work.

***Relatedness***

Some members are proactive and take it upon themselves to fulfil the need ***to belong to a community***. *‘On some mornings, I tell those that I meet that I would like to get to know new people, and those who feel the same are welcome to join me at a table later’* (Self-employed). One member mentioned community as a primary reason for joining a coworking space. *‘I could have chosen to work from home, but I felt that I wanted to be a part of a community. Otherwise, you’ll easily become quite lonely when self-employed…’* (Self-employed). Some members expressed the wish to be part of a community but were passive in fulfilling the need. There were also those who wanted deeper relationships with their peers. *‘Friendship - that’s the maximum you can achieve. Exchange numbers and hang out after work’* (Employed by large organization). However, others were indifferent all together.

How the members satisfied their need to socialize varied greatly. Some received their social interaction from the hosts: *‘Sometimes, instead of using the app to book a meeting room, I go to the reception and ask them to help me book a room, because it’s nice to have a small chat’* (Employed by large organization). It was also noted during the observations that members would often stop and chat with a host while getting a cup of coffee. Others obtained and prioritized social interaction from their colleagues at the coworking space: *‘I’m usually working at our customer’s office, so I’m only here [at the coworking space] one or two days a week, and then I prioritize socializing with my colleagues’* (Employed by large organization). There were also those who said that a sense of community was about the decorations and furniture of the coworking space.

Our findings revealed that the need to belong to a community at work varied from one individual to the next. Some large companies had multiple employees or whole departments working in the coworking space. These members interacted mainly with their own organizational colleagues. Self-employed entrepreneurs, however, had to satisfy their need for socialization by their interactions with other members or the host. The different attitudes toward the need and the ways of satisfying the need to belong to a community seemed to create tension within the coworking space and among the different members.

Related to the need to belong to a community is the need ***to have a workplace that gives you energy.*** A member said that s/he felt energized from the physical space. *‘I experience this as very fresh and a sound work environment. Everyone who works here feels that it’s a good feeling to arrive at work: a little nutrition, a vitamin kick’* (Employed by small organization). Several members said they felt energized from other members at the coworking space. *‘I’m a person that gets energized when there’s things happening around me: life and movement. I don’t necessarily need to work with them, but I need to work next to where things are happening’* (Employed by large organization). One member said that the social interactions gave him/her energy: *‘…there’s a flow of people here, coming from conferences or seminars. Many times, because of my large network, these interactions happen – “Nice to see you. How exciting. Are you working here?” and then you catch up’* (Self-employed).

***To be noticed and feel welcome at one’s workplace*** were emotions uncovered when members were asked how they felt when they entered the coworking space:

*I immediately felt that I wanted to sit here because it felt welcoming. The reception is placed at the correct spot. As soon as you open the door, you are greeted by the host who welcomes you and asks what they can help you with. There’s also a large open space that signals that everyone is welcome.* (Self-employed)

For another member, the welcome feeling was related to the host(s) in the reception: *‘It is very important that the first person you see [that works at the coworking space], like the host, is pleasant and welcoming as a person’* (Employed by large organization).

Several members said that they had high expectations of the service aspects of coworking. One member, seemingly impressed, expressed the need in the following context: *‘They [the hosts] really know how to serve their customers beyond just providing a place to work at. Sometimes they come up to you and ask if you want a coffee. It’s all these small things you know…’* (Employed by large organization).

***Autonomy***.

Some members explicitly mentioned the need ***to be in control of social interactions***. Several members said that they could show they are available for social interactions by participating in events, such as using the ping-pong tables or the social areas of the space. However, members also said that they sometimes feel torn between work and social pressure to partake in activities or events:

*They knocked on my door all the time and I love that, but then I joined, and time passed by and then I didn’t have enough time [to finish work]. Sometimes you just want to be invisible. You want to put on these glasses [refers to a picture with tinted ski-goggles] and don’t see anything or pretend as if you’re not being seen… Of course, it is fun with the community, but I believe the negative side is that you’re not in control of it.* (Self-employed)

On a similar note, a member mentioned that the space did not provide an ability to hide from others:

*‘When I come here, I know half of the people, so I can’t just go and hide. Sometimes I feel as if half of my time is used up just for saying hi to everyone. They want to tell me something, while my only wish is to pick up my computer and start working’* (Self-employed).

There were members that said it was hard to be a self-employed member in the space when there is no natural way to talk to other members. An interviewee said that this was even harder when groups of members from the same company hung out together:

*This is even more apparent in the eating area when hordes of people from other companies arrive. They might not have enough time to talk to each other during their workday. Of course, they sit together and eat just as a small business. Like many people here, you get even more lonely, which is not a problem most of the time, but some days I can feel like this…* (Self-employed).

From the observations and interviews, we found that the need for feeling in control of social interaction led to two types of tension. One tension existed among individual members who sometimes found it difficult to be in control of social interactions and they felt a social pressure to partake in social events and interactions, while they preferred to focus on their work and work alone. The other type of tension occurred between different types of members in a coworking space in which self-employed or those with small companies are interested in interaction, whereas members of larger companies seemed to be more inclined to only communicate with their own colleagues.

Another need in relation to autonomy was ***to be able to be transparent*** when meeting others. According to an interviewee working as an entrepreneur, this need was very important and amplified in a coworking environment:

*But I think that it’s difficult [to be transparent] in these kinds of places since you never know: ‘What’s your agenda? Can we have business together?’ In that case, I don’t want to be completely transparent because you don’t say to a customer, ‘Business sucks at the moment.’ Then, all chances for business with that person are gone. You only say that if you feel ‘I can trust you.’* (Self-employed).

### Business networking

Needs within business networking are about gaining direct business opportunities, building a professional network, working together with other people or companies to reach business goals, or making a good impression on guests related to the business. In general, we found that members in the smaller coworking spaces focused more on business networking.

***Relatedness***.

The need ***to have your workplace leave a good impression on guests*** was mentioned by many members. The guests are related to the companies represented in the coworking space and can be clients, potential employees, or business partners. Interviewees mentioned that the physical space and the attitude of the host(s) can leave a good impression on their company’s guest: *‘It’s a nice environment to invite potential investors and partners to. It affects how we present ourselves’* (Employed by large organization). On several occasions, the researchers observed members showing their guests around the coworking space before or after a meeting. Their guests usually seemed impressed and asked several questions about the coworking space. Several members mentioned having a positive first impression of the coworking space and the host.

Another member said that, by working at this specific coworking space, they were seen as a professional business:

*Our future suppliers or business partners will think, ‘What if we could work with this company? Then we’d be able to take our business to new heights.’ They will think we’re a serious actor doing serious things because ‘Look at where they work’* (Employed by large organization).

***Autonomy***.

The need ***to be able to market one’s business*** and ***to know who the other members are*** represent two sides of the same coin and are related to business networking. One member was asked whether s/he had the possibility to be able to control how his/her company was exposed at the coworking space.

*No, there are very few ways to do that. You can add it [the company name] to the app [the app for the coworking space], and I’ve done that. But almost no one is using the app. Other than that, there’s nothing, only your name at the entrance, and they [the coworking space provider] are rather strict in that sense, in not allowing us to market ourselves here.* (Self-employed)

When discussing the app in another interview, one member said, *‘Why should you use it? I don’t know why you’re supposed to use it. There’s a billion different apps. There has to be something that makes you take the step to download it’* (Self-employed). Later during the same interview, this member said. *‘We have customers all over Sweden. We don’t really need the exposure here. We’re not here to do business’* (Self-employed), indicating that although s/he still had the need to market his/her business, the coworking space was not a suitable venue to do so. Another member became visibly frustrated when asked whether s/he had any collaboration with other companies at the coworking space:

*Nobody knows who sits here. There’s a sign at the main entrance, and there’s just a company name. There’s nothing like a presentation once a week when a company can introduce themselves. You know all these small things. A company displays a roll-up and talks. Nothing at all. I think that’s a big downside if you’re interested in getting to know who the other companies are.* (Self-employed)

This was also confirmed by our observations, that knowing which other companies were represented by the members proved to be harder than expected. It was not until we were given access to secondary data, such as the member lists of the space, that the picture became clear.

***Competence***.

***To cooperate with relevant actors*** is all about finding partners to work with. A member expressed disappointment and frustration with their current coworking space and this need in particular: *‘The idea of these types of places is to connect people and develop cooperation. It creates a big discrepancy if my need is to experience cooperation and it’s explicitly being offered, but it still doesn’t happen’* (Self-employed). For some, cooperation is a stepping-stone to gaining business opportunities. A member was asked what relation s/he hoped to achieve with the other members: *‘Cooperate with those who sit here. Hopefully, they will become business partners. Cooperate basically’* (Employed by small organization).

Another part of networking was the explicit need ***to meet people that can lead to business opportunities.*** Interviewees stated that they hoped to be able to use new social contacts to gain business opportunities through them or their networks. Some members already had done this:

*The real purpose here is to meet other people, people who can lead to business opportunities. I’ve done it a lot here.* (Self-employed)

*I’ve gotten potential leads. One example is when I got in contact with a person that works with purchasing at [company name]. This is a potential lead for us.* (Employed by large organization).

The need to meet people that can lead to business opportunities was identified among self-employed members and those working in sales for larger corporations. But as noted by an interviewee, most individuals from larger companies did not seem to have this specific need. This could lead to tension and disappointment when fulfillment of needs between member-groups do not align:

*[After being asked what had changed during his/her membership:] The biggest difference is that these large companies, or parts of large companies have become members. And you notice a big difference…You have this company that are five people already and this other one that’s about 10–15 people. You have these large ones – they have no need to network. They think it’s nice to have a small chat, but they don’t need to network.* (Self-employed).

### Knowledge exchange

These needs are about receiving and sharing knowledge. Receiving and sharing knowledge can be related to curiosity, to giving or receiving help, or for business-related purposes.

***Competence***.

***To learn new things from peers and events*** is related to learning new things in terms of professional development or personal learning. During an interview, one member was asked what motivated him/her:

*I would say the fact that there is always something that you don’t know… I have this mentality that if you really want to know something, you can learn it. It doesn’t matter what it is. If you’re intrigued about it, you will find a way. You can ask someone [makes a hand gesture toward the space] and s/he can guide you.* (Self-employed)

A member said s/he enjoyed learning new things that were not necessarily work related:

*I’ve met people working within areas that I don’t have any clue of what it’s about, but when you sit down and talk to them you feel ‘That was exciting. I would have never known that otherwise.’ Maybe I don’t need to know it either, but it is interesting, and if you’re curious you want to learn new things.* (Self-employed)

Another member said learning new things was more related to work. *‘Seminars, lunches, they’re only 45 minutes which is optimal, you can always make room for it in your schedule. But I’ve also learnt a lot from the consultants here, for me it’s a form of professional development’* (Self-employed).

The other need related to knowledge exchange is ***to be able to receive help or input from others.*** A member said that s/he had received help and input from others several times. When asked to exemplify, s/he said,

…*we prioritize each other just because we are neighbors. I can get an assessment for free sometimes. It’s only a matter of knocking on someone’s door: ‘What do you think about this? I’m thinking about buying this for that price. Can we chat for an hour?’ They say, ‘Don’t do that’ or ‘Do this’ or ‘You’ve actually thought this through. Buy it. At that price, it’s really good.’* (Self-employed).

When the researchers were planning a workshop, two members with experience in facilitation gave valuable input that made the researchers realize they had this need as well (the workshop was canceled due to Covid-19). Another member said, *‘I’ve met several recruiting consultants here. They’ve helped me on some occasions with all the new platforms. I haven’t worked with recruiting in the last 5 or 10 years, and they can help me with it.’* (Self-employed).

On the giving end of the knowledge exchange is the need ***to be able to share knowledge.*** *‘I can contribute with something that I know because they had a problem with their computer. Then I enter their room and fix some issues with their computers and they think I’m awesome’* (Self-employed). The need can also refer to things such as helping others with personal development.

*Right now, I’m working with more ‘hard issues.’ I’d like to work more with people again, ‘softer issues.’ I want to develop organizations and people. I believe there’s a possibility of that here at the coworking space.* (Employed by small organization).

For another member, this need encapsulated both knowledge and business development. *‘It can be about conveying knowledge. For me it’s inspiring and gives me energy. It can also be more ‘hard-core,’ we [referring to other members] can develop products and services together by thinking in new ways’* (Self-employed).

### Productivity

Productivity was the largest category found and involved needs such as being able to work without distractions and being able to choose a work area that suits the work task at hand. This category also involves the need to focus on one’s core business by not having to think about, for example, cleaning the office space, enabled by the coworking space services. In general, we found that members in the larger coworking space focused more on the need for productivity.

***Autonomy***.

Observations and interviews indicated that ***to be able to focus on work activities*** was perceived as an important need. This need is related to not being disturbed by noise or questions from members or colleagues. Some members satisfied the need by working from home on some days.

*If I have to do a lot of documentation[sic], I would rather work from home, just because of the open landscape and if there is a phone call and stuff like that… often someone throws out a question. So, if I’m here, I’m not as productive as I am at home.* (Employed by large organization)

Other members solved the issue by arriving early to have some productive work hours before the majority of members showed up.

Several members mentioned the need ***to have interactions without disturbing others***. An interviewee who worked with sales said,

…*these open landscapes, if you have many phone calls and talk loudly like I do, it creates a possibility for conflict with other coworking members: people you really are supposed to be friends with. They get disturbed when you talk on the phone. Often you don’t have access to an office and then you have nowhere to go.* (Employed by large organization).

Although the coworking spaces had phone booths to let people speak on the phone without disturbing others, they were often occupied. From our observations, we noticed that on several occasions, the members were unable to find a vacant booth and had to take the phone call out in the open space or not answer the phone at all.

The aspect of productivity also incorporates the need ***to be able to choose a suitable work area.***

*When you’re trying to solve something like that [referring to a complex problem], you often go to a room and ‘whiteboard it’ out and try to solve it. I wasn’t able to do that here [compared to where s/he worked before].* (Employed by large organization)

Another member discussed his/her usage of different aspects of the coworking space. *‘I use a conference room about two or three times a week. This will probably increase as I just started out with my business… Quiet phone rooms are a good idea.* (Self-employed).

***To be able to manage confidential information securely*** was a painful point for many. One interviewee whose company had a private office said:

*We talk with companies about their future strategies and therefore we can’t sit here and spread that because someone may start to understand which company we are talking to. We have to sit separately. That is probably the hardest part. You run out all the time, back and forth, to find somewhere to take a phone call.* (Employed by large organization).

A member from a publicly traded company said,

*We have a private office and can lock our door, but to have conversations and work with papers is still risky. Absolutely no one except us can hear this information… The printer is not working as it should, so we would have to use the computer in the reception. But I would never put my USB in that computer.* (Employed by large organization).

The needs for productivity, working without disturbances, not disturbing, and dealing with confidential information sometimes created tensions with the open office and a focus on interactions and networking needs. On the one hand, members choose the co-working space for belonging to a community, interacting with other members, and building business networks, while, on the other hand, members feel a need to be productive and work alone.

***Competence***.

***To feel an increased productivity from one’s workplace*** describes how the space induces a feeling of productivity in the members. One member mentioned that s/he felt productive in the space and was asked to clarify:

*It’s about the space. This building gives me the feeling that you are working here. When you enter you have the mentality that now you’re working. It’s also contradictory because at the same time the environment is not that strict. You can sit on the couch, have a chat with someone… But still, in one hour of working here, you can deliver more than at home or at a different office.* (Employed by small organization).

***To be able to focus on the core business*** was often mentioned with the provision of various services in the coworking space. By ‘outsourcing’ mainly service tasks to the hosts, the members can focus on the activities that add value to their business.

*It simplifies everything I don’t want to do or have time to do, if you’re going to be able to live the life I’m living [refers to spending a lot of time working].* (Self-employed)

*Reception, practical things, which we didn’t have before. We used to do everything ourselves… so we save a lot of time… Now we don’t have to think about making coffee when we have guests. It’s nice that it’s always available.* (Employed by large organization).

A similar but distinct need is ***to be able to work smoothly without technical disruptions***. A member highlighted that the ability to work without having to think about any technical aspects was a positive aspect of coworking:

*‘It’s much more pleasant [comparing coworking to their old office]. Everything in addition to just having an office. I do not want to spend any time thinking about the office space. Like when the internet connection does not work, for example.* (Employed by large organization).

One interviewee said, *‘Because I’m a novice when it comes to everything technical, I feel confident that I get the help I need. To connect to the internet, use a projector, any technical aspects really’* (Self-employed).

Another member, visibly frustrated when explaining, mentioned that s/he was supposed to have a presentation, but the projector did not work with his/her computer:

*It’s all these small things [referring to the incident with the projector] that can change the perception from ‘Wow, what a nice coworking space’ to ‘This doesn’t work.’. It’s the small things that make or break the whole thing. It’s not about how the interior is designed.* (Self-employed).

### Physical well-being

***Autonomy***.

The need ***to be healthy*** was manifested in different ways. During the first visits to one of the coworking spaces, the researchers noted that a group of members went out for a run together. The host said that it was a weekly running group created by some of the members. A member said that s/he usually went to the gym during the lunch break and wished that it were in the same building: *‘Although my gym is close, it would be great if there was one in this building’* (Employed by large organization). Another member liked that his/her previous coworking space had a gym in the same building:

*I usually get a lot done after I’ve been at the gym in the afternoon. [The last coworking space] had a gym in the basement, which was a huge advantage, to be able to combine the gym with my work hour.* (Employed by large organization).

The need to be healthy came up when discussing the Covid-19 situation as well: *‘I’m worried about the coronavirus; I feel very vulnerable. As an entrepreneur, neither my family nor I can get sick, I’m worried about corona’* (Self-employed).

## Discussion

### Coworking member needs

Increasing competition in the coworking space market may call for new ways of finding competitive advantages. In this study, we uncovered 21 member needs from three coworking spaces in Sweden. The identified member needs can provide a foundation for developing new and improving existing service offerings or solutions in coworking spaces. Previous research along this line included analyzing coworking spaces’ user preferences (Weijs-Perrée et al. 2019; Appel-Meulenbroek et al. 2020). Although those findings are important with respect to coworking space research, such as highlighting the importance of accessibility and contract options, they give less insight into what coworking providers can do to improve the quality of their spaces and retain their existing members. As mentioned in Sect. 2.4, member preferences and motivations for coworking are not the same as member needs and can, according to SDT literature, even go against the basic psychological needs and hinder individual performance and well-being (cf. Deci & Ryan 2014).

The uncovered needs and five overarching categories link to the previous literature in coworking. The needs within the category of social needs can be connected to the various reasons for joining a coworking space: like the reason for social interaction (Spinuzzi 2012; Ross & Ressia 2015; Butcher 2018; Clifton et al. 2019; Weijs-Perrée et al. 2019; Appel-Meulenbroek et al. 2020), the desire to be part of a community (Spinuzzi 2012; Garret et al. 2017; Jakonen et al. 2017; Butcher 2018; Weijs-Perrée et al. 2019; Appel-Meulenbroek et al. 2020), and the wish to escape the social isolation of work (Spinuzzi 2012; Gandini 2015; Garrett et al. 2017; Jakonen et al. 2017; Clifton et al. 2019). Furthermore, the category of needs related to networking and productivity are in line with earlier research, such as networking opportunities (Gandini 2015; Ross & Ressia 2015; Brown 2017; Clifton et al. 2019; van Dijk 2019; Weijs-Perrée et al. 2019; Appel-Meulenbroek et al. 2020).

As mentioned earlier, some of the uncovered needs are in line with earlier research on preferences and motivations to join or select a coworking space. However, the innate psychological needs are more closely related to ongoing psychological growth, integrity, and well-being when the member needs are fulfilled. This study contributes to the coworking literature in gaining a deeper understanding of members’ basic needs, which are relevant for coworking space providers when they want to retain their existing, rather than potential, members.

### Tension between and within members

By uncovering the needs of coworking members, we found at least three points of tension. We refer to them as tension between the members, tension within the individual members, and tension created by privacy versus transparency issues in the space.

**Tension between the members**.

From our empirical data, we found tension among various members who have different needs for their work. When those needs are not aligned, tension can occur between the members. For example, when someone needs to take an urgent phone call or is in need of help or social interaction, this may result in disturbing other members who need to focus on their work. The tension between the members could also be attributed to the various ways the members satisfy their needs. From our findings, members who are part of larger organizations were found to have different needs than members from self-employed or smaller companies. If members primarily use the coworking space to get their work done, they might not attempt to satisfy their social needs while at work, as they rarely interacted with other members.

Specifically, some members mentioned moments of frustration when their need for autonomy in relation to being able to focus on their work was disturbed by others in the space. This is in line with the SDT literature that the need for individual autonomy might have an impact on the values of the coworking group in terms of social interaction (Deci & Ryan 2000). If the needs are satisfied, they relate to higher performance and increased well-being, but when they are not, it can lead to negative consequences (Deci & Ryan 2000). The tensions occurring in a coworking space in terms of individual autonomy and the collective needs of the coworking space could lead to individual negative consequences from an SDT perspective. This implies that the fulfilment of the basic need of autonomy on the individual level can create a tension with the collective group so that it becomes difficult in a coworking space where interaction, building up community, and networking are perceived as characteristics of coworking. The tension created between the individual versus the collective could mean in some cases that members would like to be more in control and from our data this was sometimes discussed as a reason to leave the coworking space. Tensions between different types of coworking spaces have been discussed in the literature (Bouncken et al. 2018) as well as in entrepreneurial coworking spaces (Bouncken & Reuschl 2018). However, tensions within a heterogenous coworking space, as in our study, in which different types of members are working in one space such as larger organizations as well as freelancers and small-and-medium-sized firms have been rarely discussed.

**Tension within the individual members**.

The need ‘to be in control of social interactions’ is mentioned in the coworking literature and it is stated that members have the autonomy of work and communicate to the extent and intensity they appreciate (Bouncken & Reuchel 2018). The ability to control the extent and intensity was often not present, based on our empirical data. Some members often felt torn between focusing on finishing work and being part of social interactions. On the one hand, they had the need to belong to a community and to have opportunities for social interaction. On the other hand, they also have the need to control when and how much they can interact socially. They are worried about being perceived as uninterested. This creates a dissonance of needs within an individual member, that is, tension within members.

The need to be able to focus on one’s work is connected to autonomy needs, whereas the need to belong to a community and having social interactions is connected to relatedness needs. Using the SDT lens, our data confirm that tension exists between autonomy and relatedness needs in the individual members. Specifically, Deci and Ryan (2000) wrote:

*What is dynamically interesting and is the focus of many clinical presentations is the fact that the need for relatedness can at times compete or conflict with self-organizational tendencies, that is, with the need for autonomy. Thus, much of the rich fabric of the human psyche is founded upon the interplay of the deep adaptive tendencies toward autonomy (individual integration) and relatedness (integration of the individual into a larger social whole) that are part of our archaic heritage and will, under optimal circumstances, be complementary but can, under less optimal circumstances, become antagonistic.* (p. 253)

In the coworking literature, there are some ways to tackle these internal tensions. For example, Justin (2019) has described how members use headphones to indicate that they do not want social interaction. However, Parrino (2015) mentioned that some members avoid or neglect interaction because they focus on their work and thereby are not using some of the benefits of coworking.

The relevant question for the coworking space providers is how to create a space that caters to members’ needs while simultaneously increasing the chances for needs harmony and decreasing the risks for needs dissonance. Our study adds to the research on coworking by highlighting the tensions between and within the individual members in relation to their basic needs.

**Privacy versus transparency**.

Another tension is found in terms of privacy versus openness or transparency. From the observations and interviews, some individuals feel constrained by not being able to talk freely about confidential information due to the risk of being overheard. The privacy aspect in coworking literature is recognized by other coworking researchers in relation to working in an open office space (Robelski et al. 2019; Spinuzzi 2012; Weijs-Perrée et al. 2019). To be able to focus on work activities and to be able to manage confidential information securely are often inhibited as a result of the sharing of space, creating tensions between members. However, the sharing of space is also a central enabler of other needs.

The need for privacy and reflection as well as issues with noise and concentration problems are recognized in coworking literature (Clifton et al. 2019; Weijs-Perrée et al. (2019). Emberson et al. (2010) finds evidence for decreased performance of cognitive tasks as a result from overhearing phone calls. Kim and De Dear (2013) identified distractions due to noise and loss of privacy as the major cause of workplace dissatisfaction in open-plan offices. Others mention knowledge leakage when sharing resources (cf. Waters-Lynch & Duff 2019). Even though privacy is discussed in coworking literature, the topic of confidentiality is not often taken up. Yang et al. (2019) mentioned the aspect of privacy concerns in relation to intellectual property or sensitive topics. The tension of privacy versus transparency became clear especially for the members working in larger corporations. Since coworking spaces in Sweden are increasingly used by larger firms, this tension becomes an important future focus.

***Managerial implications***.

The managerial implications of this research are mainly related to the coworking space providers. By focusing on the uncovered needs of the coworking members, coworking space providers can understand their coworking spaces’ members better. The understanding of the basic psychological needs can help coworking space providers to develop the coworking space concept and space to retain their existing members and increase their satisfaction. Making sure that those uncovered needs are met or even exceeded over time can open up various endeavors that lead to sustainable improvement work and service innovation in coworking spaces. It is also relevant that the providers are aware of the different tensions between and within the individual needs to gain an understanding of their members and how the space and coworking concept can fulfil and satisfy their members’ needs.

## Conclusions

The aim of this paper was to identify member’s basic needs in three coworking spaces in Sweden. We have uncovered, formulated, and categorized 21 member needs in relation to self-determination theory (SDT). Some needs are central to the human experience, whereas others are more work related. Tensions may arise between and within individual members. The growth of the coworking market is increasing the competition between coworking space providers, requiring new ways of finding competitive advantage. By understanding the underlying member needs and tensions in relation to these needs, coworking space providers can look for better solutions that meet or exceed their members’ needs.

One limitation of the study is a potential sample bias resulting from the three cases under study. All cases providing data in this study are similarly priced in the higher range of the Swedish coworking market. It is believed that additional complementary views could have been collected from another type of coworking space with different member characteristics.

Several avenues for future research can be explored. One area could be to study the found tensions in more detail and how members react to these tensions. For example, to what extent are the members making trade-off decisions between the needs? Another path is to try to identify the member needs at other types of coworking spaces and in other geographical locations. The generalizability of the needs must also be investigated as they could be dependent on the social context and the configuration of the coworking spaces in our study. All three are owned by large property owners located in Sweden. The aspect of prioritizing the member or customer needs is another relevant task for future research for example using the Kano model (Kano 1984).

## Electronic supplementary material

Below is the link to the electronic supplementary material.

Supplementary Material 1

Supplementary Material 2
