# Supplementary material for: In search of member needs in coworking spaces
Source: Rev Manag Sci. 2022 Apr 7;17(3):881–907. doi: 10.1007/s11846-022-00546-4 (PMC8989108; doi:10.1007/s11846-022-00546-4)
Supplement: Supplementary file 1 — Supplementary Material 1 [file 11846_2022_546_MOESM1_ESM.docx]

# **Declarations**

**Funding**: We acknowledge the financial support from CMB (Grant no.150, 'what is quality in coworking spaces') for carrying out this research.

**Conflicts of interest/Competing interests:** The authors have no conflicts of interest to declare.

**Availability of data and material:** Due to the nature of this research, participants of this study did not agree for their data to be shared publicly, so supporting data is not available.

**Code availability:** No custom code was used.

**JEL Classification codes:**

M19, O30, L85, R30
