# Supplementary material for: In search of member needs in coworking spaces
Source: Rev Manag Sci. 2022 Apr 7;17(3):881–907. doi: 10.1007/s11846-022-00546-4 (PMC8989108; doi:10.1007/s11846-022-00546-4)
Supplement: Supplementary file 2 — Supplementary Material 2 [file 11846_2022_546_MOESM2_ESM.pdf]

Adis | Bohn Stafleu van Loghum | palgrave macmillan | Springer

Change of authorship request form (pre-acceptance)

Please read the important information on page 4 before you begin

This form should be used by authors to request any change in authorship including changes in corresponding authors. Please fully complete all sections. Use black ink and block capitals and provide each author's full name with the given name first followed by the family name.

Please note: In author collaborations where there is formal agreement for representing the collaboration, it is sufficient for the representative or legal guarantor (usually the corresponding author) to complete and sign the Authorship Change Form on behalf of all authors.

Section 1: Please provide the current title of manuscript
(For journals: Please provide the manuscript ID, title and/or DOI if available.)
(For books: Please provide the title, ISBN and/or DOI if available.)

Manuscript ID no. in case of unpublished manuscript:	RMSC-D-21-00354 R2
DOI in case of published manuscript:	
ISBN (for books):	

Title: IN SEARCH OF MEMBER NEEDS IN COWORKING SPACES

Section 2: Please provide the previous authorship, in the order shown on the manuscript before the changes were introduced. Please indicate the corresponding author by adding (CA) behind the name.

	First name(s)	Family name	ORCID or SCOPUS id, if available
1st author	ERIK (CA)	RÅDMAN	0000-0002-1856-183X
2nd author	ERIK	JOHANSSON	
3rd author	HENDRY	RAHARJO	0000-0002-0420-3679
4th author	PETRA	BOSCH-SIJSTEMA	0000-0001-8141-9759
5th author			
6th author			
7th author			

Please use an additional sheet if there are more than 7 authors.

SPRINGER NATURE Springer Nature is one of the world's leading global research, educational and professional publishers, created in May 2015 through the combination of Nature Publishing Group, Palgrave Macmillan, Macmillan Education and Springer Science+Business Media.

Adis | Bohn Stafleu van Loghum | palgrave macmillan | Springer

Change of authorship request form (pre-acceptance)

Section 3: Please provide a justification for change. Please use this section to explain your reasons for changing the authorship of your manuscript, e.g. what necessitated the change in authorship? Please refer to the (journal) policy pages for more information about authorship. Please explain why omitted authors were not originally included and/or why authors were removed on the submitted manuscript.

AUTHOR 3 AND 4 WERE IN THE WRONG ORDER IN THE META DATA, HENCE NOT MATCHING THE TITLE-PAGE. THIS HAS NOW BEEN FIXED.

Section 4: Proposed new authorship. Please provide your new authorship list in the order you would like it to appear on the manuscript. Please indicate the corresponding author by adding (CA) behind the name. If the corresponding author has changed, please indicate the reason under section 3.

	First name(s)	Family name (this name will appear in full on the final publication and will be searchable in various abstract and indexing databases)
1st author	ERIK (CA)	RÅDMAN
2nd author	ERIK	JOHANSSON
3rd author	PETRA	BOSCH-SIJSTEMA
4th author	HENDRY	RAHARJO
5th author		
6th author		
7th author		

Please use an additional sheet if there are more than 7 authors.

SPRINGER NATURE Springer Nature is one of the world's leading global research, educational and professional publishers, created in May 2015 through the combination of Nature Publishing Group, Palgrave Macmillan, Macmillan Education and Springer Science+Business Media.

Adis | Bohn Stafleu van Loghum | palgrave macmillan | Springer

Change of authorship request form (pre-acceptance)

Section 5: Author contribution, Acknowledgement and Disclosures. Please use this section to provide a new disclosure statement and, if appropriate, acknowledge any contributors who have been removed as authors and ensure you state what contribution any new authors made (if applicable per the journal or book (series) policy). Please ensure these are updated in your manuscript - after approval of the change(s) - as our production department will not transfer the information in this form to your manuscript.

New acknowledgements:

NOT APPLICABLE

New Disclosures (financial and non-financial interests, funding):

NOT APPLICABLE

New Author Contributions statement (if applicable per the journal policy):

NOT APPLICABLE

State 'Not applicable' if there are no new authors.

Adis | Bohn Stafleu van Loghum | palgrave macmillan | Springer

Change of authorship request form (pre-acceptance)

Section 6: Declaration of agreement. *All* authors, unchanged, new and removed *must* sign this declaration.
(NB: Please print the form, sign and return a scanned copy. Please note that signatures that have been inserted as an image file are acceptable as long as it is handwritten. Typed names in the signature box are unacceptable.) *** Please delete as appropriate. Delete all of the bold if you were on the original authorship list and are remaining as an author.**

	First name	Family name		Signature	Affiliated institute	Date
1st author	ERIK	RÅDMAN	I agree to the proposed new authorship shown in section 4 /and the **addition/removal*of my name to the authorship list.**	[signature]	CHALMERS TEKNISKA HÖGSKOLA INSTITUTIONEN FÖR TEKNIKENS EKONOMI OCH ORGANISATION	MARCH 17 2022
2nd author	ERIK	JOHANSSON	I agree to the proposed new authorship shown in section 4 /and the **addition/removal*of my name to the authorship list.**	[signature]	CHALMERS TEKNISKA HÖGSKOLA INSTITUTIONEN FÖR TEKNIKENS EKONOMI OCH ORGANISATION	MARCH 18 2022
3rd author	PETRA	BOSCH-SIJTSEMA	I agree to the proposed new authorship shown in section 4 /and the **addition/removal*of my name to the authorship list.**	[signature]	CHALMERS tekniska högskola. inst. technology and economics	march 17 2022
4th authors	HENDRY	RAHARJO	I agree to the proposed new authorship shown in section 4 /and the **addition/removal*of my name to the authorship list.**	[signature]	CHALMERS TEKNISKA HÖGSKOLA, INSTITUTIONEN FÖR TEKNIKENS EKONOMI OCH ORGANISATION	MARCH 17 2022
5th author			I agree to the proposed new authorship shown in section 4 /and the **addition/removal*of my name to the authorship list.**			
6th author			I agree to the proposed new authorship shown in section 4 /and the **addition/removal*of my name to the authorship list.**			
7th author			I agree to the proposed new authorship shown in section 4 /and the **addition/removal*of my name to the authorship list.**			

Please use an additional sheet if there are more than 7 authors.

SPRINGER NATURE Springer Nature is one of the world's leading global research, educational and professional publishers, created in May 2015 through the combination of Nature Publishing Group, Palgrave Macmillan, Macmillan Education and Springer Science+Business Media.
